# Multilayered bacterial cellulose/reduced graphene oxide composite films for self-standing and binder-free electrode application

**DOI:** 10.1016/j.heliyon.2022.e10327

**Published:** 2022-08-18

**Authors:** Nopparut Kiangkitiwan, Thanakorn Wasanapiarnpong, Kawee Srikulkit

**Affiliations:** a Department of Materials Science, Faculty of Science, Chulalongkorn University, Bangkok, 10330, Thailand; b Center of Excellence on Petrochemicals and Material Technology, Chulalongkorn University, Bangkok, 10330, Thailand

**Keywords:** Multilayered bacterial cellulose, Bacterial cellulose/reduced graphene oxide composite film, Free-standing, and binder-free electrode

## Abstract

Multilayered bacterial cellulose (MBC)/reduced graphene oxide (rGO) composite films were fabricated using dyeing method. First, MBC films were constructed by the static culturing of kombucha SCOBY bacterial cellulose in a rectangular plastic mold for 15 days. The MBC formed on the air-liquid interface was collected and employed as the matrix for the preparation of MBC/rGO composite films using dyeing method. As found, the color strength increased with an increase in dyeing cycle due to MBC and GO (rGO precursor) affinity. However, the surface hydrophilicity was found in the opposite direction due to the restacking of hydrophobic rGO nanosheets onto MBC surface after reduction step. SEM images confirmed that MBC/rGO composite films obtained by the dyeing method exhibited the intact multilayer structure. The electrochemical behavior of free-standing and binder-free MBC/rGO electrodes was evaluated. It was found that MBC-1 exhibited the highest specific capacitance value of 192.23 F/g at the current density of 1 A/g (calculated from GCD plots) due to good diffusion of electrolyte arising from surface wettability with current density performance of 66 %. An increase in dyeing cycle (MBC-2, MBC-3, and MBC-4) led to a gradual decrease in the corresponding specific capacitance value due to a gradual increase in the electrolyte resistance derived from an increasing surface hydrophobicity of the composite films. Finally, in all cases, long-term cycle stability of more than 90 % up to 10000 cycles was achievable.

## 1 Introduction

Bacterial cellulose (BC) belongs to biomass produced by bacteria such as *Gluconacetobacter xylinus, Acetobacter xylinum*, *Komagataeibacter swingsii, Komagataeibacterrhaeticus and Komagataeibacter medellinensis* [1]. BC is a bio-nonwoven material which exhibits high purity, strength, moldability, and water-holding ability. Symbiotic culture of bacteria and yeast (SCOBY) BC is a type of BC materials that has gained scientific attention [2, 3, 4, 5]. SCOBY BC is formed on the liquid-air interface during kombucha tea fermentation in a medium containing sugar, acetic acid bacteria, and yeast. The fermentation products are mainly composed of cellulose hydrogel on air-liquid interface and acetic acid solution [6]. The unique characteristic of SCOBY BC when compared to other bacterial celluloses is that it can be cultured or constructed in the form of multilayer structure (MBC). The MBC hydrogel itself exhibits higher surface area obtained by freeze-drying or freeze-thawing techniques to prevent hornification. The SCOBY BC is easily purified by boiling in ethanol/alkali water mixture. One of interesting applications is BC composites. Graphene is an interesting material which exhibits reinforcement of soft matrixes such as BC and imparts new properties including biomedical [7], absorbents for organic pollutants [8, 9], drug delivery [10], conductive films [11, 12, 13, 14] and supercapacitors [15, 16, 17, 18, 19, 20, 21, 22, 23, 24, 25, 26, 27]. Several fabrication techniques of graphene/BC composite films including the vacuum-assisted self-assembly technique, an in-situ membrane-liquid-interface method, an in-situ cellulose bacterial culturing, direct mixing, and layer-by-layer GO/BC nanocomposite hydrogel were revealed. The particular attention was focused on conductive films and supercapacitor application. For example, a flexible, binder-free high-performance fiber-based supercapacitors was constructed by the in-situ synthesis of hierarchical polypyrrole inside the 2,2,6,6-tetramethylpiperidine-1-oxyl (TEMPO)-oxidized bacterial cellulose/reduced graphene oxide composite fiber [15]. Ti-doped FeOOH quantum dots/graphene was successfully dispersed within BC substrate as a bendable anode with large loading mass for flexible supercapacitor [16]. The fabrication of 3D porous graphene-containing nanocomposites with highly dispersed graphene nanosheets in a 3D matrix of BC by a novel layer-by-layer in situ culture method was reported [17]. The flexible nanocomposites were employed as electrodes directly without any nickel foam or stainless steel wire. The flexible holey graphene oxide/BC with a three-dimensional honeycomb structure in the presence of polyvinylpyrrolidone was prepared by bio-assembly synthesis. The composite films could be bended and twisted. The composite film was employed as free-standing electrodes [18]. The self-standing BC derived carbon/reduced graphene oxide aerogels were prepared by freeze-drying and carbonization, resulting porous carbon aerogel which exhibited excellent absorption capability and supercapacitance performance. However, both techniques are time and energy consuming methods. Several techniques of self-standing BC/rGO nanocomposite films doped with conductive polymers (polypyrrole, polythiophene or polyaniline) and a metal oxide were reported. Those techniques achieved free-standing 3D structure with excellent electrochemical performance. At present, free-standing 3D multilayered BC/rGO structure has not been reported. Therefore, in this work, self-standing multilayer BC (MBC)/reduced graphene oxide (rGO) composite films using a dyeing method were proposed.

In this research, MBC hydrogel was cultured for 15 days in a culturing medium containing sugar, peptone, yeast extract, and raw vinegar in the presence of kombucha SCOBY pellicle. Then, MBC hydrogel was collected and purified. Then, graphene oxide dispersion synthesized by Hummer's method was dyed onto dried MBC films by an exhaust dyeing method. Following that, the reduction reaction was carried out to obtain a flexible conductive MBC/rGO films. Finally, The MBC/rGO films were employed as a self-standing organic electrode to investigate electrochemical performance.

## 2 Experimental

### 2.1 Materials

SCOBY MBC hydrogel with dimension of 20” (L) x 15” (W) x 0.5” (thickness) was cultured using kombucha SCOBY starter according to our previous work [6]. Reduced graphene oxide (rGO) was synthesized from graphite by Hummer's method as described elsewhere [28].

### 2.2 Preparation of MBC/rGO composite films by dyeing method

MBC/GO composite films were obtained by exhaust dyeing using the MBC (weight):GO aqueous dispersion (volume) liquor ratio of 1:50. Four replicas were dyed separately. Each dyeing batch was continuously stirred at room temperature around 30 °C for 10 min interval. After that, the sample was taken-out and dried in open air prior to the next dyeing cycle. In this experiment, four dyed MBC/GO composite films (MBC/GO-1, MBC/GO-2, MBC/GO-3, and MBC/GO-4 which represented 5 dyeing cycles, 10 dyeing cycles, 15 dyeing cycles, and 20 dyeing cycles, respectively) were prepared. Subsequently, the films were then reduced with hydrazine hydrate for 24 h at room temperature to obtain MBC/rGO films followed by rinsing. Then, the films were dried in an oven at 60 °C for 1 h.

### 2.3 Characterizations and testings

**Figure 1:**
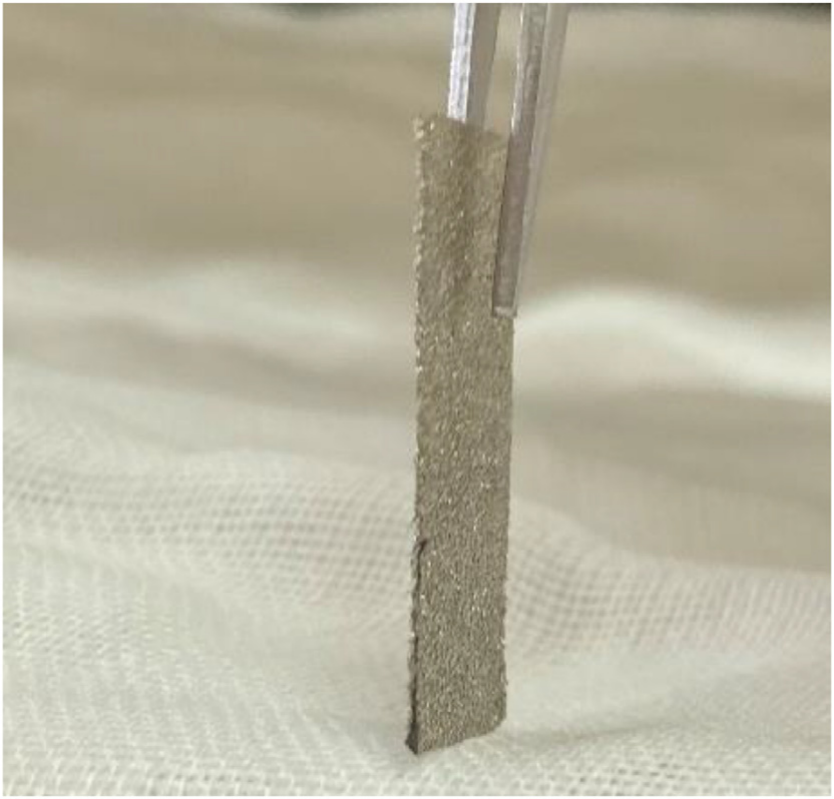
The assembly of free-standing and binder-free MBC/rGO electrode.

The morphology of the MBC film and MBC/rGO films were observed through a scanning electron microscopy (JSM-6480LV, Jeol Ltd., Japan). The cyclic voltammetry (CV) and galvanostatic charge-discharge (GCD) were evaluated by potentiostat/galvanostat instrument (Metrohm Autolab PGSTAT204). The binder-free electrode was prepared as follows (Figure 1): The sample film was sandwiched with nickel foam and compressed using a hydraulic press to maintain the dimensional stability when immersed in the alkaline electrolyte. Then, the sandwiched sample electrode was immersed in 3M KOH electrolyte for 12 h prior to the electrochemical investigation. For cyclic voltammograms, the response current densities were recorded with the potential range from 0.1 to 0.5 V (versus Ag/AgCl) at different scan rates (10, 20, 40, 60, 80 and 100 mV/s) and the specific capacitance were calculated by integrating the area under the CV curves. The galvanostatic charge-discharge was recorded at current density range of 1–10 A/g) from 0.2 to 0.45 V. The specific capacitance of the as-prepared electrode was calculated. The electrochemical impedance spectroscopy of the as-prepared electrode samples was performed with open circuit potential using HIOKI IM3590 chemical impedance analyzer from 0.01 Hz to 100 kHz with alternate current amplitude of 10mV. The cycle stability was investigated by potentiostat/galvanostat instrument (Metrohm Autolab, PGSTAT204, Netherlands). The free-standing and binder-free MBC/rGO electrode was fabricated for the GCD cycle stability which was tested at 1 A g^−1^ from 1 to 10 000 cycles in 3 M KOH electrolyte.

## 3 Results and discussion

### 3.1 Physical appearance, wettability, and electrical conductivity

**Figure 2:**
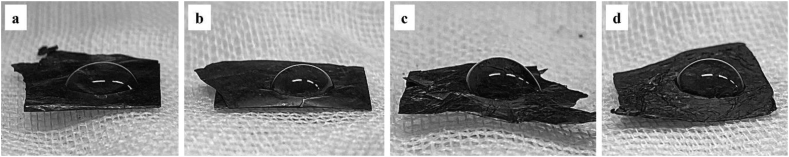
Physical appearance and wettability (observed after 10 s) of (a) MBC-1, and (b) MBC-2, (c) MBC-3, and (d) MBC-4. Note that the water droplets slowly disappeared by time.

**Figure 3:**
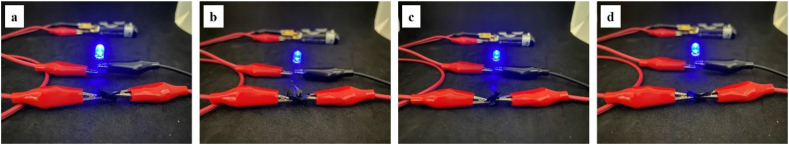
LED circuit connection of (a) MBC-1, (b) MBC-2, (c) MBC-3 and (d) MBC-4.

The physical appearance of MBC-1, MBC-2, MBC-3, and MBC-4 samples and their corresponding water wettability are represented in Figure 2. As seen, the color strength of the composite films increases with an increase in a dyeing cycle, indicating that each and every dyeing shifts the dyeing equilibrium towards the MBC The rGO weight gain is found in the following order; MBC-1 (17.95% ± 0.12) < MBC-2 (23.53% ± 0.10) < MBC-3 (27.88% ± 0.15) < MBC-4 (33.89% ± 0.15). The finding results are found consistent with the surface wettability which gradually decreases with an increase in a dyeing cycle due to the precipitation of restacking hydrophobic rGO nanosheets (the graphitic structure) onto MBC surface, resulting in hydrophobic surface as shown in Figure 2. To evaluate the electrical conductivity of MBC/rGO composite films, LED circuit was connected as shown in Figure 3. The results indicate that MBC/rGO composite films are electrically conductive, implying that rGO nanosheets are evenly absorbed into MBC as well as precipitated onto MBC surface.

### 3.2 SEM analysis

**Figure 4:**
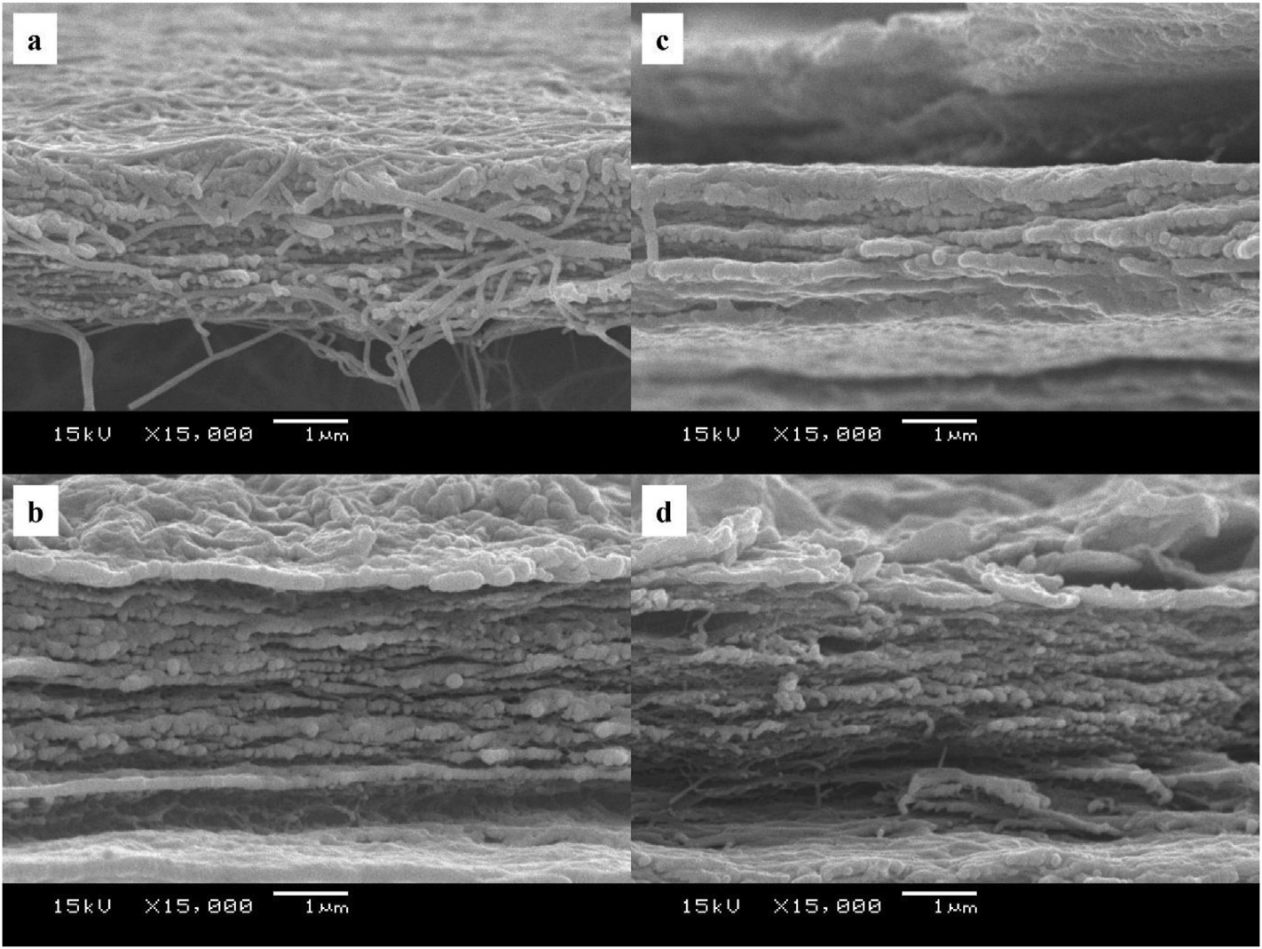
SEM images (cross-sectional view) of (a) MBC, (b) MBC-1, (c) MBC-2 and (d) MBC-4.

SEM cross-sectional images of MBC, MBC-1, MBC-2, and MBC-4 samples are compared as shown in Figure 4. As seen, MBC (which is the cell wall of bacterial) exhibits the non-woven structure composed of multilayers. In case of the MBC/rGO composite films, filaments were covered with rGO due to the intermolecular hydrogen bonding interaction at the interface of rGO hydroxyl groups and cellulose hydroxyl groups as reported in our previous work [6]. As a result, MBC/rGO composite films exhibited an increase in Young's modulus values with an increase in dyeing cycle as the following order; MBC < MBC-1 < MBC-2 < MBC-3 < MBC-4 due to the reinforcement effect of rGO nanosheets. The achievable MBC/rGO composite films are foldable which are suitable for flexible supercapacitor electrode.

### 3.3 Electrochemical performance of self-standing and binder-free MBC/rGO composite films

**Figure 5:**
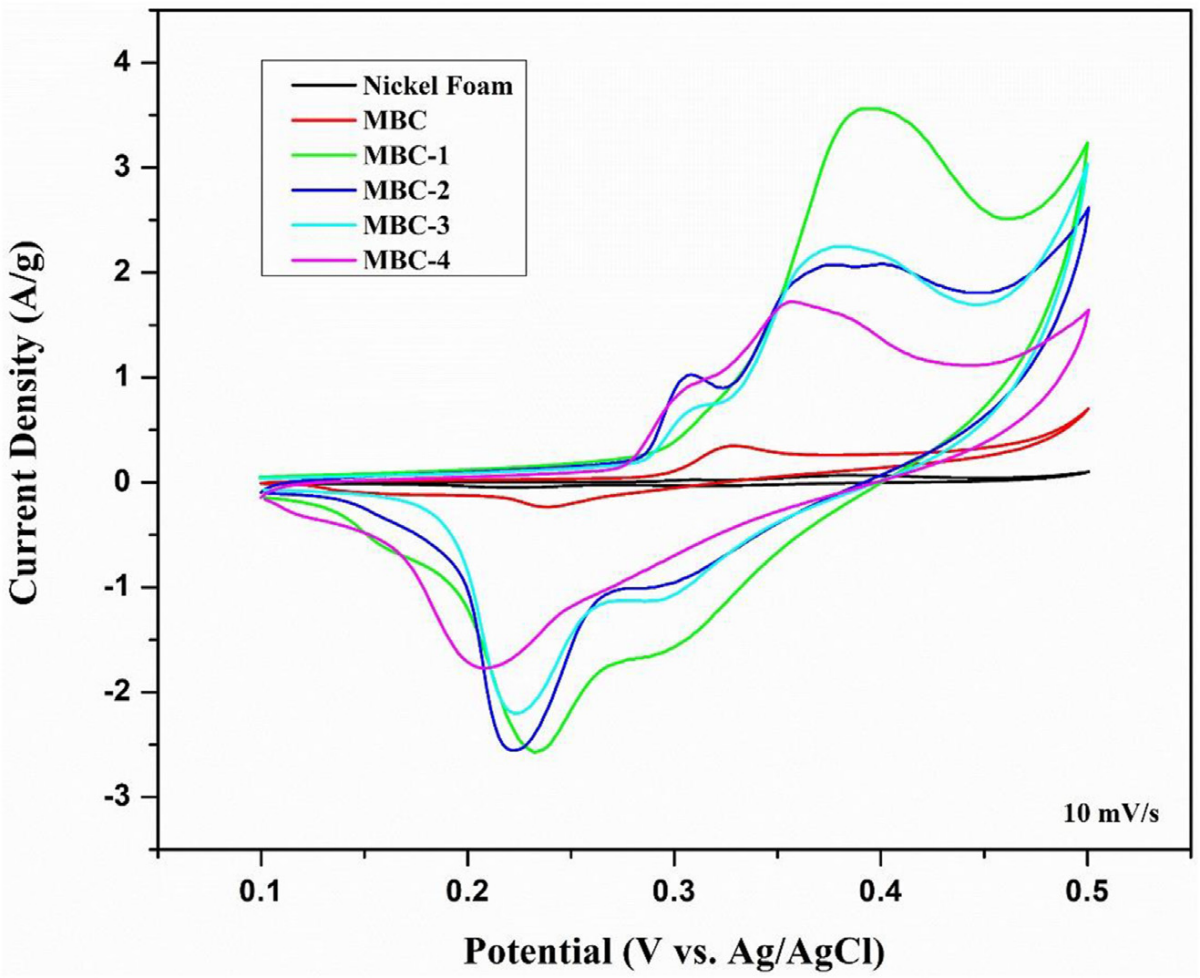
CV plots of MBC/rGO composite films (MBC-1, MBC-2, MBC-3 and MBC-4).

**Scheme 1:**
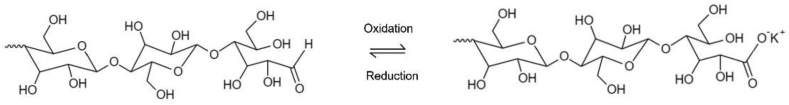
The oxidation/reduction of BC.

The self-standing and binder-free MBC/rGO electrodes were fabricated as explained in Section 2.3. The cyclic voltammetry (CV) and galvanostatic charge-discharge (GCD) were measured. Cyclic voltammograms are plotted between the current at working electrode and applied voltage. Figure 5 illustrates the CV plots of nickel foam, MBC, MBC-1, MBC-2, MBC-3, and MBC-4 using voltage ranging from 0.1-0.5 V at a scan rate of 10 mV/s. Note that the specific capacitance value (5 F/g) of bare nickel foam under the same condition is negligible, implying that nickel hydroxide is not formed at this condition. All CV plots represent pseudocapacitance behavior judged by distorted shapes which show the redox peaks corresponding to the oxidation/reduction of cellulose as shown in Scheme 1 [29].

The specific capacitance values are calculated as shown in Table 1. At a scan rate of 10 mV/s, MBC, MBC-1, MBC-2, MBC-3, and MBC-4 exhibit the specific capacitance value of 32.18, 234.90, 175.45, 170.28, and 138.85 F/g, respectively. The specific capacitance values decrease with an increase dyeing cycle due to the reduction of surface hydrophilicity and surface area, resulting in an increase in the electrolyte resistance. During the reduction step, GO hydrophilic groups (hydroxyl, epoxide, and carboxylic groups) are converted back to sp^2^ double bonds, resulting in the irreversible restacking of nanosheets with decreasing surface area combined with increasing hydrophobicity. As a result, the electrolyte resistance builds up at electrolyte-electrode interface, resulting in poor diffusion ability of the electrolyte. Generally, at a low scan rate the electrolyte diffusion rate to the electrode is high which results in fast diffusion of ions from the solution to deposit on the electrode surface, leading to high surface adsorption/desorption of ions. An increase in a scan rate results in poorer adsorption/desorption performance. Consequently, the capacitance value decreases with an increase in a scan rate, implying the hydrophobic characteristic of the electrode material.

**Table 1:** Specific capacitance values of MBC/rGO composite films (MBC-1, MBC-2, MBC-3, and MBC-4).

Sample	Specific Capacitance (F/g)					
	10 mV/s	20 mV/s	40 mV/s	60 mV/s	80 mV/s	100 mV/s
Nickel Foam	5.04	-	-	-	-	-
MBC	32.18	24.40	21.09	19.82	19.27	18.93
MBC-1	234.90	226.88	215.50	209.33	204.19	199.25
MBC-2	175.45	158.00	147.44	142.42	138.75	135.93
MBC-3	170.28	157.00	152.19	149.58	146.97	144.13
MBC-4	138.85	124.10	112.63	106.17	101.41	97.20

**Figure 6:**
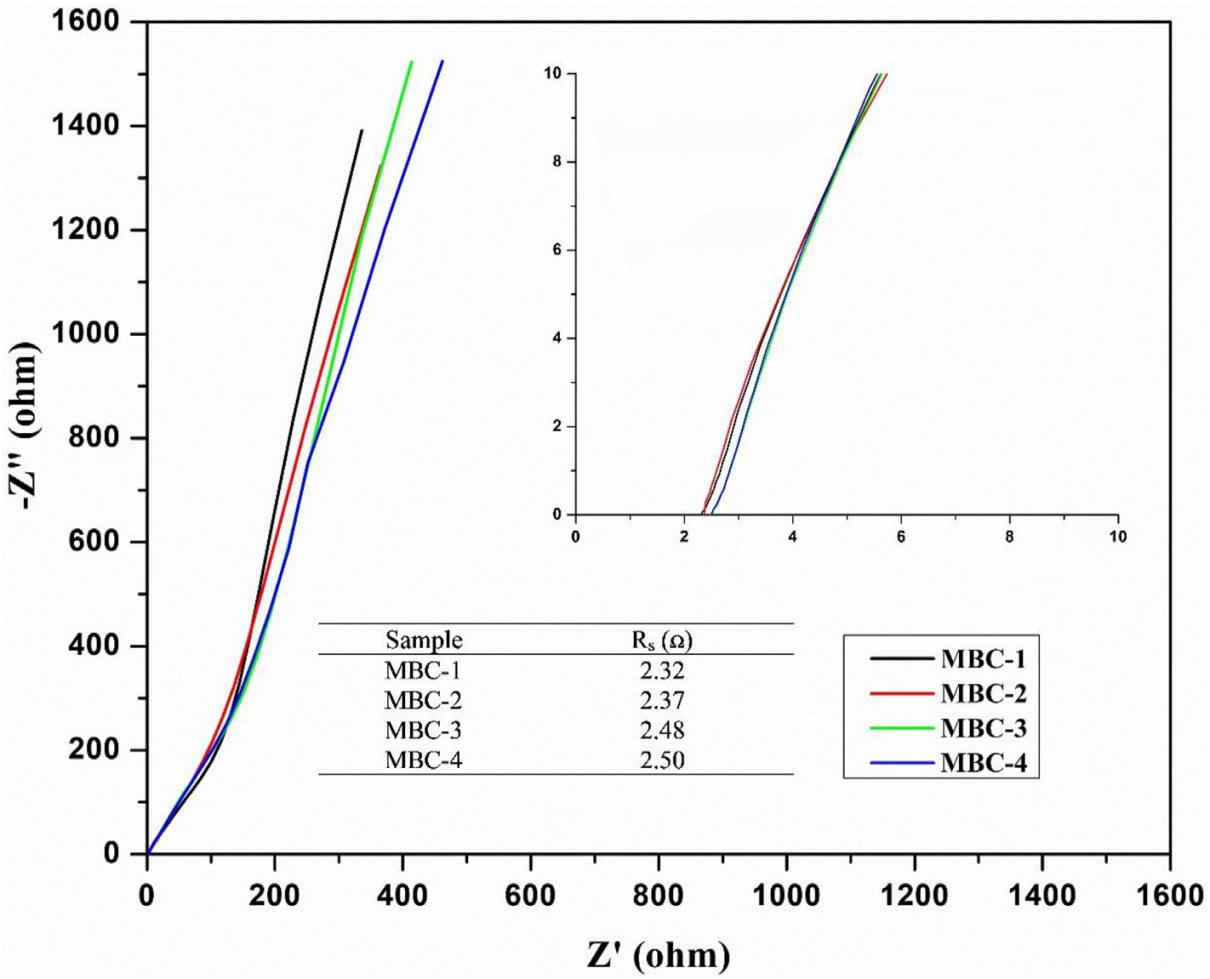
Nyquist plots at frequency range 0.01–100 kHz for the as-prepared composite electrodes (MBC-1, MBC-2, MBC-3, and MBC-4).

To further confirm the electrolyte resistance, the electrochemical impedance spectroscopy (EIS) at open circuit potenal in the frequency range 0.01–100 kHz was carried out [34, 35]. The Nyquist plots are shown in Figure 6. The uncompensated solution resistance (R_S_) including of electrolytic resistance, the internal resistance, and the contact resistance at the electrolyte-film interface was determined at the x-axis interception. The R_S_ values of MBC-1, MBC-2, MBC-3, and MBC-4 are found to be 2.32, 2.37,2.48, and 2.50 Ω, respectively which correspond well with the explanation of adsorption/desorption performance.

**Figure 7:**
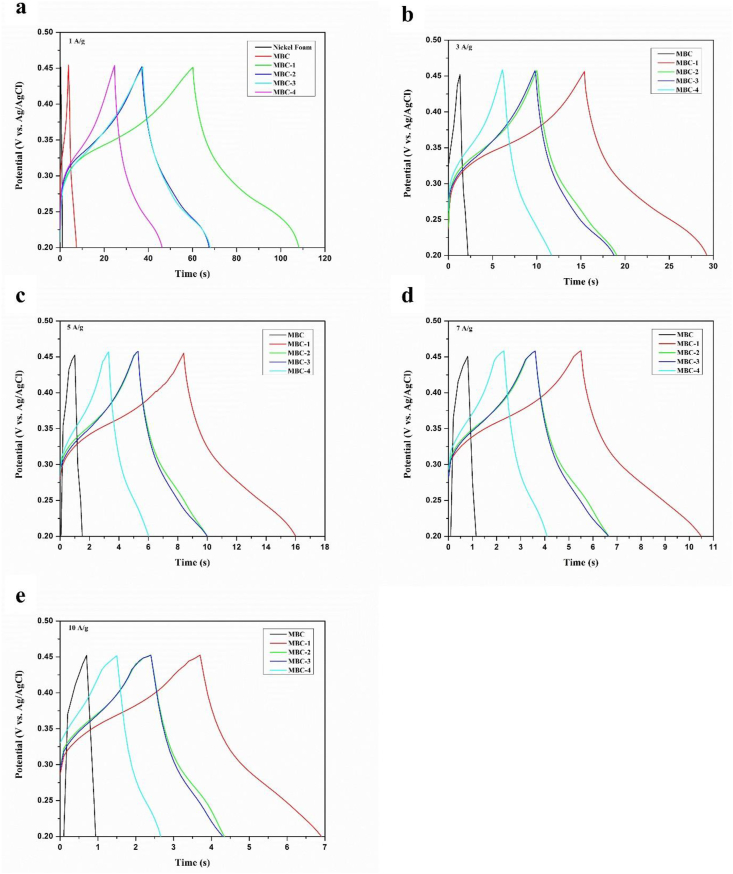
GCD plots of MBC/rGO composite films at different current densities (a) 1 A/g, (b) 3 A/g, (c) 5 A/g, (d) 7 A/g and (e) 10 A/g.

**Figure 8:**
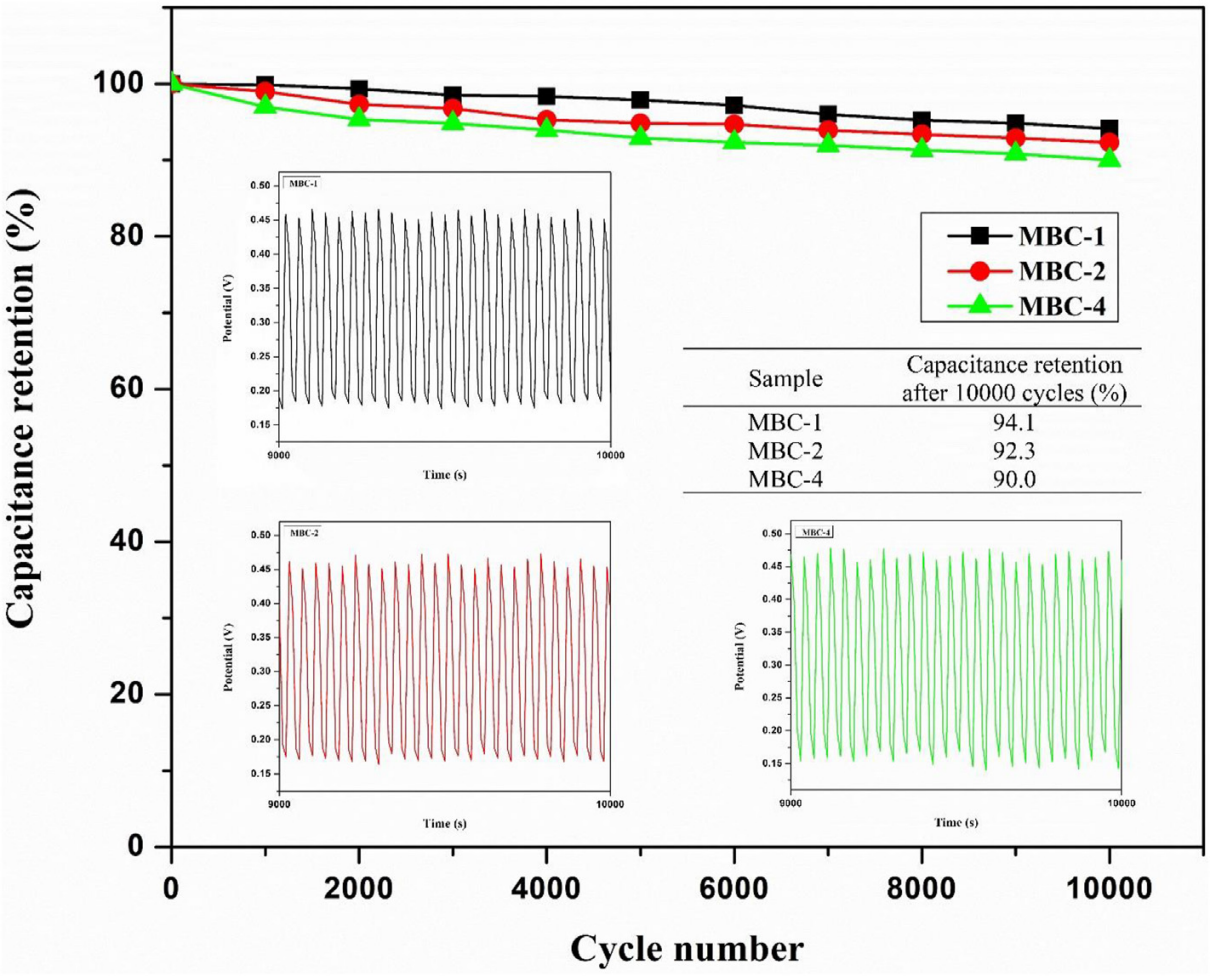
Long-term cycle stability of the as-prepared electrodes (MBC-1, MBC-2, and MBC-4) after 10000 cycles.

Galvanostatic charge-discharge (GCD) measurement was carried out to investigate the intrinsic capacitance capability at current densities of 1 A/g, 3 A/g, 5 A/g, 7 A/g, and 10 A/g (Figure 7). All GCD curves exhibit slightly distorted triangular shapes, resembling the shape of electrical double-layer capacitance from rGO and pseudocapacitance from MBC. It is observed that the discharging time decreases in the following order: MBC-1 > MBC-2 > MBC-3 > MBC-4 > MBC, indicating that capacitance retention decreases with an increase in dyeing cycle as summarized in Table 2. It is found that specific capacitance values decrease in the similar manner to those calculated from CV curves. For an example, at current density of 1 A/g, MBC-1, MBC-2, MBC-3, MBC-4, and MBC exhibit specific capacitance values of 192.23, 122.12, 121.98, 86.34, and 14.54 F/g, respectively. These results indicate that MBC-1 performs the highest capacitance performance due to its lowest solution resistance against electrolyte adsorption. The solution resistance is inversely proportional to surface area and hydrophobicity; the lower the surface area the higher the solution resistance and the higher the hydrophobicity the higher the solution resistance. The current density performance at least 47.46 % and more is achieved which are indicative of supercapacitor efficiency. It is recommended that an anti-restacking agent should be added during dyeing to prevent the problem of rGO nanosheet restacking. The long-term cycle stability of the as-prepared electrodes are shown in Figure 8, demonstrating that the capacitive retention of MBC-1, MBC-2, and MBC-4 can be preserved more than 90% after 10000 cycles at current density of 1 A/g.

**Table 2:** Specific capacitance values of MBC/rGO composite films (MBC-1, MBC-2, MBC-3, and MBC-4) and their corresponding current density performance calculated from GCD plots.

Sample	Specific Capacitance (F/g)					Current Density Performance (%)				
	1 A/g	3 A/g	5 A/g	7 A/g	10 A/g	1 A/g	3 A/g	5 A/g	7 A/g	10 A/g
MBC	14.54	11.04	10.62	9.09	6.24	100	75.91	73.02	62.53	42.93
MBC-1	192.23	166.27	150.91	138.14	127.37	100	86.50	78.51	71.86	66.26
MBC-2	122.12	107.19	95.22	85.01	77.77	100	87.77	77.97	69.61	63.69
MBC-3	121.98	106.08	95.25	85.27	78.03	100	86.97	78.09	69.90	63.97
MBC-4	86.34	64.03	55.34	49.12	40.97	100	74.16	64.09	56.89	47.46

Finally, the electrochemical performance including specific capacitance value and long-term stability is compared with other works as presented in Table 3.

**Table 3:** The comparative electrochemical performance of the MBC/rGO electrode with other reports.

Electrode materials	Electrolyte	Current or Scan rate	Specific capacitance (F/g)	Long term cycles	Reference
MBC/rGO	3 M KOH/3 electrodes	1 (A/g)	192.23	94.1%/10000	This work
HRGO/BC	PVA-H_3_PO_4_/2 electrodes	0.4 (A/g)	65.9	88.0%/2000	[18]
BC/GO	1 M H_2_SO_4_/3 electrodes	0.4 (A/g)	160	90.3%/2000	[27]
PPY/RGO/BC	1.0 M NaNO_3_/3 electrodes	2 (mA/cm^2^)	235.2	64.7%/5000	[25]
PANI/BC/GN	1 M H_2_SO_4_/3 electrodes	1 (mA/cm^2^)	477	56.3%/8000	[26]
PPY/BC/RGO	1.0 M NaNO_3_/3 electrodes	1 (mA/cm^2^)	271	73.5%/8000	[22]
Co_3_O_4_/GN/BC	2 M KOH/3 electrodes	3 (mA/cm^2^)	1274.2	96.4%/20000	[21]
PPy@TOBC/rGO	1 M H_2_SO_4_/3 electrodes	0.5 (A/g)	391	79.0%/5000	[15]
PPY/BC	2.0 M LiCl/3 electrodes	2 (mA/cm^2^)	216.4	94.5%/5000	[30]
BC-MWCNTs-PANI	1 M H_2_SO_4_/3 electrodes	1 (A/g)	656	99.8%/1000	[31]
PPy/CuO/BC	2 M NaCl/2 electrodes	0.8 (mA/cm^2^)	601	64.1%/300	[32]
N,P-CNWs from BC	6 M KOH/3 electrodes	1 (A/g)	258	98.0%/30000	[33]
Ni(OH)_2_–H/RGO/BC	2 M KOH/3 electrodes	5 (mA/cm^2^)	877.1	93.6%/15000	[23]
PPy/rGH-PSS	1 M H_2_SO_4_/3 electrodes	1 (A/g)	640.8	90.0%/2000	[34]
PPy/CoS/BC	2 M NaCl/2 electrodes	0.7 (A/g)	614	62.4%/300	[36]

## 4 Conclusions

In this experiment, the multilayered BC (MBC) obtained from SCOBY kombucha tea fermentation was employed as a matrix for the preparation of MBC/rGO composite films using dyeing method. GO (rGO precursor) exhibited excellent affinity to MBC arising from intermolecular hydrogen bonding force. After reduction reaction, reduced graphene oxide (rGO) was achieved, resulting in the flexible conductive MBC/rGO films confirmed by LED circuit illumination. The surface wettability of MBC/rGO composite films notably decreased in the following order; (MBC-4 < MBC-3 < MBC-2 < MBC-1) due to the restacking of hydrophobic rGO nanosheets onto the MBC surface. The electrochemical behavior of free-standing and binder-free MBC/rGO electrodes was evaluated. It was found that MBC-1 exhibited the highest specific capacitance value of 192.23 F/g at the current density of 1 A/g (calculated from GCD plots) due to good diffusion of electrolyte arising from surface wettability. An increase in dyeing cycle (MBC-2, MBC-3, and MBC-4 led to a gradual decrease in the corresponding specific capacitance value due to a gradual increase in an electrolyte resistance derived from an increasing surface hydrophobicity as confirmed by EIS analysis. Therefore, it is important that hydrophilicity modification of MBC/rGO composite films is necessary in order to improve the specific capacitance value. Finally, long-term cycle stability up to 10000 cycles showed that capacitance retention of 90% was achievable.

## Declarations

### Author contribution statement

Nopparut Kiangkitiwan: Performed the experiments.

Thanakorn Wasanapiarnpong: Analyzed and interpreted the data.

Kawee Srikulkit: Conceived and designed the experiments; Analyzed and interpreted the data; Contributed reagents, materials, analysis tools or data; Wrote the paper.

### Funding statement

This work was supported by the 90th Anniversary of 10.13039/501100002873Chulalongkorn University, Rachadapisek Sompote Fund (GCUGR1125633059D) and 10.13039/501100017170TSRI Fund (CU_FRB640001_01_62_1).

### Data availability statement

Data included in article/supplementary material/referenced in article.

### Declaration of interests statement

The authors declare no conflict of interest.

### Additional information

No additional information is available for this paper.
